# Has COVID-19 Affected the Course of Chickenpox in Children?

**DOI:** 10.3390/v16121912

**Published:** 2024-12-13

**Authors:** Justyna Franczak, Zuzanna Wasielewska, Anna Maria Fotyga, Krystyna Dobrowolska, Justyna Moppert, Małgorzata Sobolewska-Pilarczyk, Małgorzata Pawłowska

**Affiliations:** 1 Department of Infectious Diseases and Hepatology, Collegium Medicum, Bydgoszcz, Nicolaus Copernicus University, 87-100 Torun, Polandmpawlowska@cm.umk.pl (M.P.); 2 Department of Paediatrics, Infectious Diseases and Hepatology, Voivodeship Infectious Observation Hospital, 85-030 Bydgoszcz, Poland; 3 Collegium Medicum, Jan Kochanowski University, 25-317 Kielce, Poland

**Keywords:** chickenpox, children, COVID-19

## Abstract

Objectives of the study: The aim of this study was to assess the impact of the COVID-19 pandemic on the epidemiology and clinical course of chickenpox in children based on 6 years of self-reported observations. MATERIAL AND METHODS: The medical records of 350 patients under 18 years of age hospitalised in the Department of Paediatrics, Infectious Diseases, and Hepatology between 1 January 2018 to 31 December 2023 were analysed retrospectively. RESULTS: During the analysed period, 350 children were hospitalised due to chickenpox, the fewest in the pandemic period, the greatest number in 2023. Complications of chickenpox were diagnosed in 297 children (84.86%). The most commonly diagnosed complications were bacterial dermatitis (168, 48%) and lower respiratory tract infections (13.42%). After the pandemic period, a generalised inflammatory reaction was observed significantly more often (*p* ≤ 0.01069). Among children hospitalised in 2023, 5.90% of patients with bacterial dermatitis required surgical intervention due to skin abscess or fasciitis. In 2023, 41.46% of cultures from swabs taken from skin lesions grew *Streptococcus pyogenes*. After the pandemic, children with chickenpox and gastrointestinal symptoms were hospitalised significantly less frequently (*p* ≤ 0.00001). CONCLUSIONS: In the post-pandemic period, there were more hospitalisations of patients with chickenpox complicated by bacterial skin infection progressing to a generalised inflammatory reaction or septicaemia, related to the increase in invasive group A streptococcal infections observed in Europe. On the other hand, thanks to the widespread adaption of hygiene practices and infection prevention measures, the number of patients with gastrointestinal symptoms significantly decreased.

## 1. Introduction

Chickenpox is one of the most common infectious diseases of childhood caused by the varicella-zoster virus (VZV). The source of infection is a sick person; the gateway to infection is the upper respiratory tract. The virus is transmitted by the droplet route and with air movement over distances of up to several tens of metres. [1,2]. The risk of contracting the disease after direct contact with a household member with chickenpox is 80–90%. The incubation period of chickenpox is 10–21 days. The infectious period begins 2 days before the rash appears and lasts until all lesions have dried up (about 6 days). In Poland, up to 200,000 cases of chickenpox are reported annually among children and adults. Preschool and early school-age children are most often ill, adults much less frequently. The highest incidence is typically observed in winter and spring [2,3,4].

Primary VZV infection has a benign, self-limiting course in most cases. However, it can lead to serious complications requiring hospitalisation, such as secondary bacterial infections of the skin and soft tissues, respiratory tract infections, and neurological complications. Vaccinations effectively prevent chickenpox infections and, consequently, reduce the risk of complications associated with the disease [1,5].

The COVID-19 pandemic caused a wide range of serious health, social, and economic consequences. To counteract the pandemic, various measures and restrictions have been put in place, such as lockdowns, closures, social distancing, hygiene, and protective measures such as wearing face masks. It can be suggested that countermeasures to prevent human-to-human transmission also successfully led to a demonstrated reduction in cases of influenza, whooping cough, measles, mumps, scarlet fever, and chickenpox during the pandemic [6,7]. These measures may have suppressed chickenpox transmission, lowering herd immunity, potentially creating an ‘immunity debt’, especially in countries without universal chickenpox vaccination. A long period of low exposure to pathogens has resulted in a significant reduction in protective immunity and increased susceptibility to infections in a large part of the paediatric population, which is associated with an increased risk of developing epidemics of infectious diseases. This may have been the reason for the increased incidence of chickenpox after the pandemic. Chickenpox infection usually occurs in early childhood. As a result of the COVID-19 pandemic, chickenpox virus infection may shift to adolescence or adulthood, during which chickenpox infections are associated with more severe consequences. The widespread collective immunity to chickenpox induced by the vaccine may have mitigated the effects of the ‘immunity debt’. However, the COVID-19 pandemic reduced the level of routine childhood vaccinations [8].

The SARS-CoV-2 (Severe Acute Respiratory Syndrome Coronavirus 2) pandemic reached Poland in March 2020, leading to the closure of preschools, schools, universities, and the country’s borders. A state of epidemic was officially declared from 20 March 2020 to 15 May 2022, as per the Ministry of Health’s regulations [9].

According to the current literature, it can be assumed that the majority of children in the population likely contracted COVID-19 during this period [10]. It appears that the period of isolation and the outbreak of SARS-CoV-2 may have influenced the clinical picture of chickenpox.

## 2. Objective of the Study

The objective of this study was to evaluate the impact of the COVID-19 pandemic on the epidemiology and clinical course of chickenpox in children using the example of children hospitalised in the Department of Paediatrics, Infectious Diseases, and Hepatology between 2018 and 2023.

## 3. Materials and Methods

The medical records of 350 patients under 18 years of age hospitalised from 1 January 2018 to 31 December 2023 in the Department of Paediatrics, Infectious Diseases, and Hepatology with a diagnosis of chickenpox (primary or secondary diagnosis as defined by the International Classification of Diseases ICD-10) were analysed retrospectively. The following parameters were analysed: age and sex, number of children with chickenpox complications, type of complications, length of hospitalisation, coexisting immune disorders and chronic diseases, and type and length of treatment.

Three time periods were conventionally distinguished:

Group I—patients hospitalised in 2018–2019, i.e., in the period before the COVID-19 pandemic.

Group II—patients hospitalised between 2020 and 2021, i.e., during the COVID-19 epidemic and increased social isolation.

Group III—patients hospitalised between 2022 and 2023, i.e., in the period after the COVID-19 outbreak.

Continuous data including age, CRP (C-reactive protein) level, time from onset of the disease to hospitalisation, length of hospitalisation, and duration of treatment with antivirals and antibiotics were assessed with the Shapiro–Wilk test. As a result of non-Gaussian distribution, the data were described using the median and interquartile range (IQR), while the differences between groups were evaluated using the nonparametric Mann–Whitney test. The Bonferroni correction was applied due to multiple comparisons. Categorical data were described using numbers and percentages. Group comparisons were conducted using Pearson’s χ2 test or Fisher’s exact test depending on the group size. Statistical significance was defined as *p*-values of less than 0.05. All statistical analyses were performed using Statistica v. 13 (StatSoft, Tulsa, OK, USA).

## 4. Results

**Figure 1:**
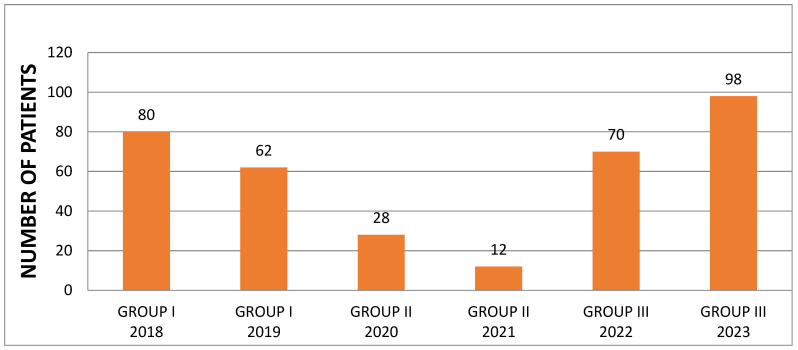
Number of hospitalised patients in different years.

During the analysed period, 350 patients with a clinical diagnosis of chickenpox were hospitalised in the Department of Paediatrics, Infectious Diseases, and Hepatology. Among them, there were 166 girls and 184 boys. On average, 58 children were hospitalised annually, with the lowest number during the pandemic period (28 children in 2020, 12 children in 2021) and the highest in 2023, with 98 admissions (Figure 1).

There were 142 patients in the identified group I (hospitalised in 2018–2019); 40 patients in group II (hospitalised in 2020–2021); and 168 patients in group III (hospitalised in 2022–2023).

Patients’ ages ranged from 1 month to 18 years, with a median age of 4 years (Table 1). Infants accounted for 23.14% of patients (81 children), with a median age of 6 months. There were 123 children in the age range 1–3 years (35.14%); 4–6 years—96 children (27.43%); 7–10 years—32 children (9.14%); and 11–18 years—18 children (5.14%). The length of hospitalisation ranged from 1 to 21 days, with a median hospitalisation of 5 days (Table 2). There were no statistically significant differences in patient age or length of hospitalisation between the study groups.

There were 93 children (26.60%) with chronic diseases, the most common of which were bronchial asthma and allergic diseases, found in 53 children (15.14%). Immune disorders were present in seven children (2.30%). In most children, the source of chickenpox virus infection was unknown (60%—210 children), before the pandemic significantly more often than in the post-pandemic period (69.72% vs. 52.38%). In 110 children (31.70%), it was another infection in the family, while 29 children (8.30%) had contact with chickenpox in educational institutions (nursery, kindergarten, school).

Compared to the pre-pandemic period, the time from the onset of symptoms to hospitalisation after the pandemic was significantly prolonged (3.5 days in Group I, 4 days in Group III) (Table 1).

Prior to hospital admission, 66 children (19%) were treated causally for an average of 3 days. During hospitalisation, 312 children (89.14%) were treated with antiviral drugs, including 111 with intravenous acyclovir, 77 with oral acyclovir, and 124 with sequential treatment (initially intravenous, then oral). The median duration of antiviral treatment was 7 days. There was no statistically significant difference in the use of antiviral treatment in the pre- and post-pandemic periods. Antibiotic therapy was included in 261 children (74.57%), systemic therapy in 247 children, and local antibiotic therapy only in 13 children; the median treatment was 8 days. Before the pandemic, antibiotic therapy was administered in 96 children (68.09%), during the pandemic period in 34 children (85%), and, in the post-pandemic period, in 128 children (78.92%) (Table 2). In the post-pandemic period, systemic therapy was used significantly less frequently alone and more often in combination with local antibiotic therapy. There was no statistically significant difference in the duration of antibiotic therapy before and after the pandemic.

Fever was observed in 254 subjects (72.60%), with a median duration of 3 days. In group I, fever was observed in 102 children (71.83%), median duration 3 days; in group II, fever was observed in 30 patients (75%), median duration 2 days; in group III, fever was observed in 122 patients (72.62%), median duration 3 days. The CRP concentration on admission averaged 20.73 mg/L, median 7.1. Significantly higher CRP concentrations were observed in post-pandemic children. In group I, the value of this inflammatory index averaged 18.08 mg/L, median 5.7; in group II, 15.46 mg/L, median 5.2; and, in group III, 24.06 mg/L, median 8.5. (*p* ≤ 0.006852, Table 1). There was no statistically significant difference between the incidence of cough before and after the pandemic. However, after the pandemic, concurrent diarrhoea was observed statistically significantly less frequently—21.28% in group I vs. 4.76% in group III (*p* ≤ 0.00001, Table 1).

**Figure 2:**
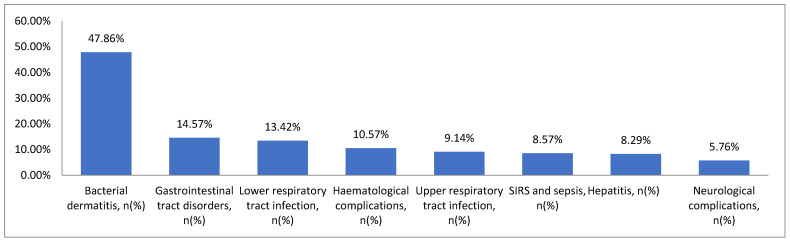
The frequency of chickenpox complications in children hospitalised between 2018 and 2023.

Complications of chickenpox occurred in 297 children (84.86%). The most frequently diagnosed complications were bacterial dermatitis (168, 47.86%) and respiratory tract infections. Upper respiratory tract infections were observed in a total of 9.14% of the cases (32 children), while pneumonia and bronchitis occurred in 47 children (13.42%). Gastrointestinal disorders—vomiting, loose stools, abdominal pain—occurred in 51 children (14.57%). Neurological complications were found in 20 children (5.76%). Among them, acute cerebellar ataxia was found in 10 children (2.86%). Haematological disorders were observed in 37 (10.57%) children, most frequently leukopenia with neutropenia and thrombocytopenia (16 children, 4.57%). Of the less common complications, arthritis was observed in eight children (2.29%), otitis media in seven children (2%), and hepatitis in twenty-nine children (8.29%) (Figure 2).

**Figure 3:**
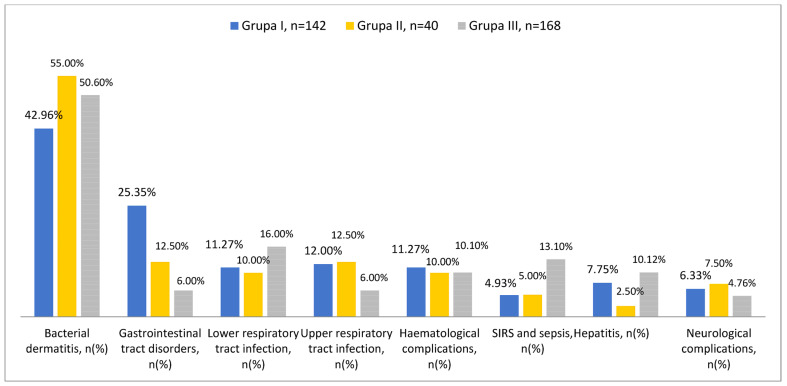
The frequency of chickenpox complications divided into groups: Group I (children hospitalised in 2018 and 2019—blue); Group II (children hospitalised in 2020 and 2021—orange); and Group III (children hospitalised in 2022 and 2023—grey).

In group I—in the period before the COVID-19 pandemic—the most common complication of chickenpox was bacterial dermatitis—61/142 (42.96%). Neurological complications (seizures, convulsions, cerebellar ataxia) occurred in 9/142 patients (6.33%). Gastrointestinal symptoms were reported by 36/142 children (25.35%). Upper respiratory tract infections were observed in 17/142 (12%) patients and pneumonia in 16/142 children (11.27%). Haematological complications occurred in 16/142 (11.27%) children, including thrombocytopenia in five children (3.52%). Hepatitis occurred in 11/142 children (7.75%), while otitis media and arthritis occurred in two children (1.20%). A generalised inflammatory reaction was diagnosed in 5/142 (3.52%) children, while septicaemia was diagnosed in two children (1.20%) (Figure 3).

In group II, the most common complication was bacterial dermatitis—22/40 (55%). Neurological complications (seizures, convulsions, cerebellar ataxia) occurred in 3/40 patients (7.50%). Gastrointestinal symptoms were reported by 5/40 children (12.5%). Upper respiratory tract infections were observed in 5/40 patients (12.50%) and pneumonia in 4/40 children (10%). Haematological complications occurred in 4/40 children (10%), including thrombocytopenia in one (2.50%). Hepatitis and otitis media occurred in one child (2.50%). A generalised inflammatory reaction occurred in two children (5%) (Figure 3).

In group III, the most common complication was bacterial dermatitis—85/168 (50.60%). Neurological complications (seizures, convulsions, cerebellar ataxia) occurred in 8/168 patients (4.76%), including cerebellar ataxia in four children (2.38%). Gastrointestinal symptoms were reported by 10/168 children (6%). Upper respiratory tract infections were observed in 10/168 (6%) patients and pneumonia in 27/168 children (16.0%). Haematological complications occurred in 17/168 (10.12%) children, including thrombocytopenia in 10 (5.95%). Hepatitis occurred in 17/168 children (10.12%). Arthritis occurred in six children (3.57%). A generalised inflammatory reaction was diagnosed in 19/168 (11.31%) children and septicaemia in three children (1.78%). Five patients with bacterial skin infection in 2023 required surgical intervention due to abscess formation (5.90%) (Figure 3).

Comparing the incidence of chickenpox complications in the different groups, before the pandemic, bacterial dermatitis and subcutaneous tissue inflammation were less common (group I—42.96% vs. group II—55% and group III—50.60%), while, after the pandemic, generalised inflammatory reaction was diagnosed significantly more often—more than threefold (3.52% in group I vs. 11.31% in group III) (Table 3). In the group of children hospitalised in 2023, 5.90% of patients with bacterial dermatitis required surgical intervention due to skin abscesses or fasciitis. In 2023, 41.46% (17/41) of the skin swab cultures grew *Streptococcus pyogenes*, while 21.95% (9/41) grew *Staphylococcus aureus*. Comparing respiratory tract infections after the pandemic, children with features of upper respiratory tract infection during chickenpox were hospitalised less frequently (Group III—6% vs. Group I—12%), while pneumonia was observed slightly more frequently (Group III—16.07% vs. Group I—10%). After the pandemic, children with chickenpox and gastrointestinal symptoms were hospitalised significantly less frequently (Group III: 6% vs. Groups II and I: 25.35% and 12.50%, respectively). There were also slightly fewer neurological complications (group I: 6.33% vs. group III: 4.76%). However, hepatitis was observed more frequently (Group III: 10.12% vs. Groups II and I: 7.75% and 2.50%, respectively). Haematological disorders occurred with a similar frequency in all groups.

## 5. Discussion

The above analysis is a continuation of a retrospective study conducted at our centre in 2018, which included 761 patients hospitalised for chickenpox in the Department of Paediatrics, Infectious Diseases, and Hepatology between 1 January 1999 and 31 December 2017. Comparing the average annual number of hospitalisations for chickenpox since 1999, we have seen a higher number of hospitalisations in recent years —58 cases from 2018 vs. 40 cases per year until 2017. The highest number of hospitalised patients in the last 25 years was recorded in 2023, with 98 children. However, the length of hospitalisation has not changed since 1999; the median in both surveys was 5 days, and the median age of patients was 4 years. Children under 5 years of age accounted for 65% of patients until 2017, and, in the study covering 2018–2023, 70.85% of children were under 5 years of age. The most common complications of chickenpox until 2017 were respiratory tract infections (30.10%) (12.10% upper respiratory tract infections, 18% pneumonia and bronchitis) followed by bacterial skin infections in 24.80% of children, then gastrointestinal symptoms—18.60%. However, as of 2018, among complications, bacterial skin infection was the most frequently diagnosed—almost twice as often—in 47.86%. Symptoms of respiratory tract infections (9.14% upper respiratory tract infections and 13.42% pneumonia and bronchitis) and gastrointestinal symptoms (14.57%) were slightly less frequent. Haematological complications occurred with a similar frequency (in 1999–2017—9.10% vs. in 2018–2023—10.57%) [5].

In a study of children hospitalised for chickenpox at the Provincial Hospital for Infectious Diseases in Warsaw in 2019 and 2022, the most common complication was bacterial superinfection of skin lesions, which was found in 70.6% of cases (in our study in 55% of children in 2022 and 50.6% in 2023). Connective tissue inflammation and septicaemia were more common (as in our study), the use of antibiotics increased (from 71.3% to 85.2%), and a combination of two antibiotics was more often required at the same time. More patients were admitted in a severe general condition, fever persisted longer, and the length of hospitalisation increased (5 days in 2022 vs. 4 days in 2019). A lower incidence of neurological complications was observed (4.3% in 2022 vs. 8.5% in 2019) [11]. Our study confirms the observed trends, although a significantly more severe course of bacterial dermatitis with generalised inflammatory reaction and the need for surgical intervention was observed in children hospitalised in 2023.

According to the authors of another analysis, the burden of hospital complications due to chickenpox in Poland between 2006 and 2021 appeared to be higher than the average reported by other EU countries. The majority of complications were in children under 9 years of age, the age group with the highest prevalence of chickenpox infections [12]. The most common complications of chickenpox were dehydration (15.9%), skin, and soft tissue infections (14.6%), pneumonia (12.2%), and cerebellitis (11%) [13].

As indicated by a number of scientific studies, the COVID-19 pandemic affected the epidemiology of infectious diseases, which showed a decreasing trend in the early phase of the COVID-19 era. The above trend was also noticeable in our study, with a 72% decrease in the number of patients hospitalised with chickenpox between 2020 and 2021. This was influenced by infection prevention measures against COVID-19 (school closures, observance of social distance, widespread masking, and hand hygiene). A study in Japan involving 3.5 million children with chickenpox and herpes zoster between 2005 and 2022 observed two changes in the trend of chickenpox incidence, the first after the introduction of vaccines into the routine immunisation programme in 2014 and the second during the COVID-19 outbreak, when there was a 57.2% reduction in chickenpox incidence, a 65.7% reduction in antiviral use, and a 49.1% reduction in healthcare costs [14].

Attention is also drawn to the decrease in the frequency of gastrointestinal complications in our study, a trend that is likely also associated with increased hygiene measures and changes in behaviours related to medical care. The most important measures in preventing faecal–oral transmission are hand hygiene and disinfection, proper sanitation, food safety, and surface disinfection. Masks and protective gloves used when handling food help prevent contamination with bacteria or viruses. Protective clothing effectively shields the skin from direct contact with waste or contaminated surfaces. Masks, on the other hand, primarily protect against respiratory pathogens. Their main purpose is to limit the spread of droplets containing viruses, which are emitted during coughing, sneezing, talking, or breathing. Similar conclusions were reached by Moshe et al., who provided an overview of the impact of the COVID-19 pandemic on the picture of paediatric infectious diseases. According to the authors, the pandemic led to changes in the circulation patterns of respiratory pathogens, including influenza, RSV (Respiratory Syncytial Virus), and *Streptococcus pneumoniae*, and reduced transmission rates of urinary tract infections and gastrointestinal infections [15].

The Japanese authors, who conducted a survey of 3417 patients hospitalised with infectious diseases in 18 hospitals in Japan from July 2019 to June 2022, observed a significant reduction in RSV infections after the spread of COVID-19 in 2020 and small numbers of children hospitalised with this cause until March 2021. They then observed an unexpected out-of-season RSV outbreak in August 2021 (50 patients per week), particularly evident among older children aged 3–6 years. An increase in infections with norovirus aetiology was also observed in April 2021, suggesting that COVID-19 risk-reducing nonpharmaceutical interventions were less effective against norovirus and RSV. Influenza, human metapneumovirus infections, *Mycoplasma pneumoniae*, and rotavirus gastroenteritis were rarely observed for more than 2 years [16].

The COVID-19 pandemic had a significant and multifaceted impact on the childhood infectious disease picture, while SARS-CoV-2 infection alone resulted in mild symptoms and a favourable prognosis in children. These included persistent symptoms of SARS-CoV-2 infection among children (‘long COVID’), changes in healthcare delivery (particularly through widespread adoption of telemedicine), and changes in circulation patterns of various pathogens, including influenza, RSV, and *Streptococcus pneumoniae*, as well as viruses causing gastrointestinal infections. Decreased vaccination coverage and increased vaccination hesitancy were due to disruption of routine vaccination programmes. In addition, the pandemic was associated with aspects of antibiotic overuse [17].

Fascciola et al. analysed infectious disease notifications in the three-year pre-pandemic period (2017–2019) and the pandemic period (2020–2022) in the territory of the province of Messina, Italy. They showed that the total number of notifications decreased significantly by 41% during the pandemic period compared to the pre-pandemic period, with a very large reduction in the number of cases of diseases such as measles and chickenpox. The authors concluded that a number of factors, including social constraints and hygiene adherence, reduced the risk of contracting infections transmitted, particularly by the droplet route. At the same time, they noted the risk of underreporting due to the burden on health professionals, as there was a significant percentage decrease in the reporting of certain infectious diseases during the pandemic period compared to the pre-pandemic period [18].

**Figure 4:**
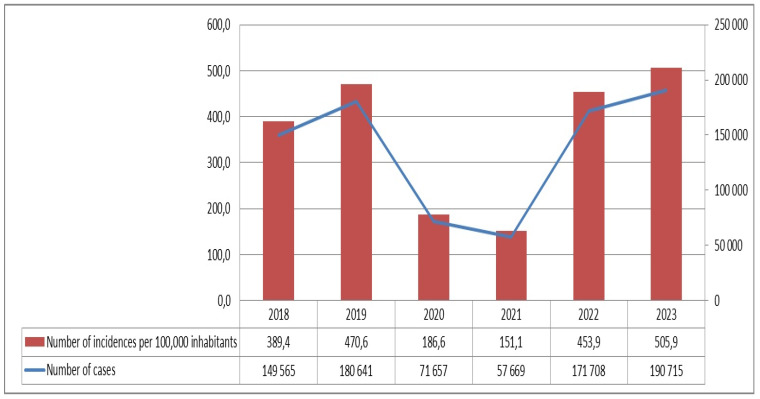
Number of cases and incidences per 100,000 inhabitants of chickenpox in Poland in different years.

In Poland, we could also see a significant reduction in the incidence of chickenpox in 2020 and 2021. In 2020, 71,567 children were affected by chickenpox; the incidence was 186.6 per 100,000 population. Of the children, 0.51% required hospitalisation (368 patients). Children aged 0–4 years were most commonly ill (36,661—51%). In 2021, 57,669 children were ill; the incidence was 151.1 per 100,000 population. There were 210 children hospitalised, representing 0.36%. In contrast, significantly more children were ill before the pandemic. In 2018, 149,565 children contracted chickenpox, 0.73% required hospitalisation (1089 patients), and the incidence was 389.4 per 100,000. A total of 72,797 patients were children aged 0–4 years (48.7%). In 2019, 180,641 were ill, 0.64% were hospitalised (1156 children), and the incidence was 470.6 per 100,000. From 2022, a renewed increase in incidence was observed, with 171,708 children, 839 were hospitalised (0.49%), and the incidence was 453.9 per 100,000 population. Children aged 0–4 years accounted for 45% (77,836). In contrast, the highest number of children became ill in 2023—190,715; the incidence was 505.9 per 100,000 population. There were 0.57% of children hospitalised due to complications (1084 persons) (Figure 4) [4,19].

In 2023, the Department of Paediatrics, Infectious Diseases, and Hepatology recorded the highest number of hospitalisations for chickenpox since 1999—98 children. One of the reasons for the increased number of hospitalisations of children with chickenpox in 2023 was the increase in *Streptococcus pyogenes* infections observed across Europe [20]. On 2 December 2022, a warning was first published in the UK regarding an increase in *Streptococcus pyogenes* infections, in addition to tonsillitis, scarlet fever, and invasive infections (iGAS). Viral infections, such as chickenpox, predispose to invasive iGAS infections [21]. Bacterial skin infections with concomitant generalised inflammatory reaction, septicaemia, fasciitis, and toxic shock syndrome were observed significantly more frequently in our patients with chickenpox in 2023, which was observed much less frequently before the pandemic. In 2023, a significant proportion of our patients with bacterial skin infection in the course of chickenpox had *Streptococcus pyogenes* cultured in smears (41.46% of samples taken from skin lesions).

Reports of an increase in iGAS cases compared to pre-COVID-19 pandemic levels have been observed simultaneously in many European countries, including the United Kingdom, France, Sweden, Ireland, Denmark, and Spain, as well as on other continents. In the study, the authors reported a significant rise in the total number of group A Streptococcus (GAS) cases in the paediatric population in Houston, Texas, starting in October 2022. In 2022, a total of 318 individual GAS cases were identified. Children aged 0 to 4 years were affected most frequently (45/101 [44.6%]) [22]. The authors of another study also describe this trend. Beginning in October 2022, several states across the United States, including Colorado, Minnesota, and Texas, observed unusual rises in paediatric iGAS cases. The median age among patients was 5.7 years; 66% were boys, and 70% of patients had no underlying health conditions [23].

In 2022, a sevenfold increase in invasive *Streptococcus pyogenes* (iGAS) infections in children aged 0–5 years was observed in the Netherlands compared to the years before the COVID-19 pandemic. Of the 42 cases in this age group, seven had previous or coincident chickenpox infections, and nine were fatal. The authors suggest that the increases in iGAS incidence in children may be attributed to a larger group of susceptible children resulting from reduced exposure to GAS and/or other predisposing infections such as chickenpox, hemiplegia, and other respiratory viruses at times of adherence to social distancing during the COVID-19 pandemic. After 2 years of low incidence, the recurrence of predisposing viral infections may have exacerbated the recurrence of iGAS infections [24].

The Spanish PedGas-Net Research Group, a multicentre network including 51 Spanish hospitals for the study of iGAS, analysed trends in iGAS infections in Spain, finding a significant increase in infections in late 2022 and early 2023, which was greater compared to the years before the pandemic. A hypothesis was put forward that the immune response of children may have been weakened by isolation measures taken during the pandemic, with an associated lack of exposure to infectious agents. This ‘immunity debt’, which would account for the large pool of susceptible individuals, may also have played a role in the increased number of all viral and bacterial infections in numbers far exceeding those of previous years [25,26,27].

The term “immunity debt” was first used by Cohen et al. in 2021 in the context of the COVID-19 pandemic and refers to the phenomenon in which the population, due to isolation and other measures aimed at preventing the spread of the virus, did not have the opportunity to be exposed to various pathogens that would normally be present in society. As a result, the immune system did not have the chance to “train”, making people more susceptible to other infections once the restrictions were lifted [28].This phenomenon affects all countries that experienced strict restrictions in response to COVID-19, but it is most noticeable in countries that had long-term lockdowns, such as the United Kingdom, Italy, Spain, France, the United States, Canada, and Australia. It is worth emphasising that this term may apply not only to COVID-19 but also to other global health crises that lead to prolonged social isolation and reduced interpersonal contact.

The documented impact of SARS-CoV-2 infection on the immune system through modulation of the cellular response alters the course of common childhood diseases such as chickenpox. According to ECDC reports, there was an increase in a number of infectious diseases after the pandemic, including infections caused by parvovirus B19, *Mycoplasma pneumoniae*, and *Streptococcus pyogenes*, as a consequence of changes in the immune system [29]. Further research is needed to understand the impact of SARS-CoV-2 infection on immune response mechanisms.

## 6. Conclusions

The COVID-19 pandemic period had a major impact on the course of infectious diseases in children. In the post-pandemic period, there were more hospital admissions of patients with chickenpox complicated by bacterial skin infection progressing to a generalised inflammatory reaction or septicaemia, which was related to the increase in invasive group A streptococcal infections observed in Europe. On the other hand, thanks to the spread of hygiene habits and infection prevention measures, the number of patients with gastrointestinal symptoms decreased significantly. The COVID-19 pandemic has affected the epidemiology of chickenpox, and further research is needed to better understand its long-term effects. This will provide insights that can help better prepare for similar health crises in the future.

## Figures and Tables

**Table 1:** Baseline characteristics of patients hospitalised in three time periods due to varicella infection.

Parameter	Pre-Pandemic Period (A) n = 142	Pandemic Period (B) n = 40	A vs. B *p*-Value	Post-Pandemic Period (C) n = 168	A vs. C *p*-Value	B vs. C *p*-Value
Sex, girls/boys, n (%)	73 (51.41)/69 (48.59)	10 (25)/30 (75)	0.00297	83 (49.4)/85 (50.6)	0.62576	0.01403
Age in years, median (IQR), max–min	4 (2–6), 1–15 years n = 107	4 (1.5–5), 1–17 n = 31	0.608069	4 (2.5–6), 1–17 n = 131	0.616425	0.386893
Age in months median (IQR), max–min	5.6 (3.3), 0–11 months n = 35	6 (2–7), 1–9 n = 9	0.626593	6 (3–9), 0–11 n = 37	0.973206	0.478136
Comorbidities						
Any, n (%)	36 (25.35)	5 (12.5)	0.08568	52 (30.95)	0.27589	0.01870
Bronchial asthma, n (%)	6 (4.23)	1 (2.5)	0.52067	7 (4.17)	0.59871	0.52358
Allergies, n (%)	5 (3.52)	0 (0)	0.28460	4 (2.38)	0.39644	0.42263
Immune disorders, n (%)	2 (1.41)	0 (0)	0.60780	6 (3.57)	0.20333	0.27282
Atopic dermatitis, n (%)	8 (5.6)	2 (5)	0.61779	18 (10.71)	0.04728	0.21688
History of contact			0.32558		0.00519	0.84344
Unknown, n (%)	99 (69.72)	23 (57.5)		88 (52.38)		
Home, n (%)	36 (25.35)	13 (32.5)		61 (36.31)		
Educational institution (nursery, preschool, school)	6 (4.23)	4 (10)		19 (11.31)		
Hospital, n (%)	1 (0.7)	0 (0)		0 (0)		
Time from onset to hospitalisation (in days), median(IQR), min–max	3.5 (2–5), 0–10	4 (2–5), 0–10	0.376708	4 (3–6), 0–17	0.002334	0.005577
Symptoms at baseline						
Fever, n (%)	102 (71.83)	30 (75)	0.02693	122 (72.62)	0.87729	0.76029
Nausea, vomiting, n (%)	41 (29.08) n = 141	5 (12.5)	0.02328	31 (18.45)	0.02776	0.26034
Diarrhoea, n (%)	30 (21.28) n = 141	4 (10.26) n = 39	0.08818	8 (4.76)	0.00001	0.16971
Cough, n (%)	38 (26.76)	10 (25.64) n = 39	0.88844	44 (26.19)	0.90973	0.94388
Laboratory parameters at baseline						
CRP (mg/L), median (IQR), max–min	5.7 (1.6–17.8), 0–289.4	5.2 (1.6–15.5), 0.1–80.4	0.090051	8.5 (2.85–27.45), 0.6–280.5	0.006852	0.110459

Abbreviations: IQR: interquartile range; CRP: C-reactive protein.

**Table 2:** Length of hospitalisation and treatment used during hospitalisation due to varicella infection in three time periods.

Parameter	Pre-Pandemic Period (A) n = 142	Pandemic Period (B) n = 40	A vs. B *p*-Value	Post-Pandemic Period (C) n = 168	A vs. C *p*-Value	B vs. C *p*-Value
Length of hospitalisation in days, median (IQR), min–max	5 (4–7), 1–16 n = 141	5 (4–6.5), 2–21	0.310820	5 (4–7), 2–20	0.532402	0.156186
Antiviral treatment with acyclovir during hospitalisation	127 (89.44)	36 (90)	0.91802	149 (88.69)	0.83409	0.81239
Intravenous, n (%)	46 (36.22)	13 (36.11)	0.98331	52 (35.14) n = 148	0.96208	0.99265
Oral, n (%)	30 (23.62)	9 (25)		37 (25) n = 148		
Switched from oral to intravenous, n (%)	51 (40.16)	14 (38.89)		59 (39.86) n = 148		
Time of antiviral treatment in days, median (IQR), min–max	7 (5–7), 2–16 n = 127	6 (4–7), 2–10 n = 36	0.103235	7 (6–8), 3–14 n = 149	0.074154	0.008421
Treatment with antibiotics, n (%)	96 (68.09) n = 141	34 (85)	0.03583	131 (78.92) n = 166	0.03121	0.38695
Systemic, n (%)	83 (86.46)	27 (79.41)	0.29385	91 (70) n = 130	0.00604	0.54160
Local, n (%)	5 (5.21)	1 (2.94)		7 (5.38) n = 130		
Switched from systemic to local, n (%)	8 (8.33)	6 (17.65)		32 (25) n = 130		
Time of antibiotic treatment in days, median (IQR), min–max	8 (7–10), 3–14 n = 96	7 (7–8), 2–11 n = 34	0.073693	8 (7–10), 2–27 n = 131	0.764448	0.045133

Abbreviations: IQR: interquartile range.

**Table 3:** Varicella complications observed in children hospitalised during three time periods.

Parameter	Pre-Pandemic Period (A) n = 142	Pandemic Period (B) n = 40	A vs. B *p*-Value	Post-Pandemic Period (C) n = 168	A vs. C *p*-Value	B vs. C *p*-Value
Complications						
Any, n (%)	121 (85.21)	35 (87.50)	0.71482	141 (83.93)	0.61902	0.75103
Bacterial dermatitis, n (%)	61 (42.96)	22 (55)	0.15238	85 (50.60)	0.17952	0.61641
Lower respiratory tract infection, n (%)	16 (11.27)	4 (10)	0.54076	27 (16.07)	0.22277	0.24107
Hepatitis, n (%)	11 (7.75)	1 (2.50)	0.78022	17 (10.12)	0.46778	0.10239
SIRS, n (%)	5 (3.52)	2 (5)	0.47933	19 (11.31)	0.01069	0.18727
Sepsis, n (%)	2 (1.41)	0 (0)	0.60780	3 (1.79)	0.57877	0.52509
Neurological complications						
Acute cerebellar ataxia n (%)	4 (2.82)	1 (2.50)	0.69706	5 (2.98)	0.60356	0.67451
Haematological complications						
Leukopenia, neutropenia, n (%)	13 (9.15)	2 (5)	0.31726	6 (3.57)	0.03540	0.47642
Thrombocytopenia, n (%)	5 (3.52)	1 (2.50)	0.60618	10 (5.95)	0.23489	0.33832
Anaemia, n (%)	3 (2.11)	1 (2.50)	0.63291	4 (2.38)	0.59198	0.66024

Abbreviations: SIRS, systemic inflammatory response syndrome.

## Data Availability

Data supporting the reported results can be provided upon request from the corresponding author.
